# Aflatoxin Reduction in Maize by Industrial-Scale Cleaning Solutions

**DOI:** 10.3390/toxins12050331

**Published:** 2020-05-17

**Authors:** Michelangelo Pascale, Antonio F. Logrieco, Matthias Graeber, Marina Hirschberger, Mareike Reichel, Vincenzo Lippolis, Annalisa De Girolamo, Veronica M. T. Lattanzio, Katarina Slettengren

**Affiliations:** 1 National Research Council of Italy, Institute of Sciences of Food Production (CNR-ISPA), 70126 Bari, Italy; antonio.logrieco@ispa.cnr.it (A.F.L.); vincenzo.lippolis@ispa.cnr.it (V.L.); annalisa.degirolamo@ispa.cnr.it (A.D.G.); veronica.lattanzio@ispa.cnr.it (V.M.T.L.); 2 Bühler AG, 9240 Uzwil, Switzerland; matthias.graeber@buhlergroup.com (M.G.); marina.hirschberger@buhlergroup.com (M.H.); 3 Eurofins WEJ Contaminants, 21079 Hamburg, Germany; MareikeReichel@eurofins.de

**Keywords:** grain cleaning, maize, aflatoxins, remediation, mechanical cleaning, optical sorting

## Abstract

Different batches of biomass/feed quality maize contaminated by aflatoxins were processed at the industrial scale (a continuous process and separate discontinuous steps) to evaluate the effect of different cleaning solutions on toxin reduction. The investigated cleaning solutions included: (i) mechanical size separation of coarse, small and broken kernels, (ii) removal of dust/fine particles through an aspiration channel, (iii) separation of kernels based on gravity and (iv) optical sorting of spatial and spectral kernel defects. Depending on the sampled fraction, dynamic or static sampling was performed according to the Commission Regulation No. 401/2006 along the entire cleaning process lines. Aflatoxin analyses of the water–slurry aggregate samples were performed according to the AOAC Official Method No. 2005.008 based on high-performance liquid chromatography and immunoaffinity column cleanup of the extracts. A significant reduction in aflatoxin content in the cleaned products, ranging from 65% to 84% with respect to the uncleaned products, was observed when continuous cleaning lines were used. Additionally, an overall aflatoxin reduction from 55% to 94% was obtained by combining results from separate cleaning steps. High levels of aflatoxins (up to 490 µg/kg) were found in the rejected fractions, with the highest levels in dust and in the rejected fractions from the aspirator and optical sorting. This study shows that a cleaning line combining both mechanical and optical sorting technologies provides an efficient solution for reducing aflatoxin contamination in maize.

## 1. Introduction

Maize is one of the most important food crops in the world and it is a staple food for the majority of sub-Saharan African countries. However, it is a cereal that is largely susceptible to mycotoxin contamination, causing serious health risks to humans and animals, particularly in developing countries [1,2,3]. Among mycotoxins that can contaminate the maize, aflatoxins (AFs) are considered of most concern due to their toxic effects on humans and animals. Aflatoxins are secondary metabolites produced primarily by *Aspergillus flavus* and *A. parasiticus* with aflatoxin B_1_, B_2_, G_1_ and G_2_ (AFB_1_, AFB_2_, AFG_1_ and AFG_2_) more frequently occurring in a variety of foods including maize, peanuts, dried fruits, spices and tree nuts, as well as feedstuffs. The presence of AFB_1_ in feedstuffs intended for dairy animals poses an additional risk for humans due to its transference into milk and dairy products with the presence of aflatoxin M_1_ (AFM_1_), a product of AFB_1_ metabolism. Aflatoxins are potent liver carcinogens (hepatocellular carcinoma) and are mutagenic, teratogenic, hepatotoxic and immunosuppressive, causing acute and chronic human health disorders [4]. For example, aflatoxin-contaminated maize has been implicated in the past in acute aflatoxicosis outbreaks in rural Kenya, resulting in a large number of illness cases and deaths [5,6]. The International Agency for Research on Cancer (IARC) classifies AFs (naturally occurring mixtures of AFB_1_, AFB_2_, AFG_1_, AFG_2_ and AFM_1_) as Group 1 human carcinogens [7]. In order to protect human and animal health, maximum permitted levels for AFs in several commodities and feed have been fixed, both at European and international level. For instance, in the European Union, for maize (and rice) to be subjected to sorting or other physical treatment before human consumption, a limit of 5 µg/kg (AFB_1_) and 10 µg/kg (sum of B_1_, B_2_, G_1_ and G_2_) has been established [8].

In a global context, aflatoxin contamination is considered a real concern in all tropical and subtropical regions. However, in recent years, due to climate change, AFs have also become an increasing problem in countries where aflatoxin contamination was previously not a risk. For example, high levels of AFs were found in maize intended for feed in southeast Europe during the 2012–2013 cropping season [9,10]. To prevent and reduce mycotoxin contamination in cereals and other commodities, several codes of practice and guidelines have been published [11,12,13]. They include Good Agricultural Practices (GAP) and Good Storage and Manufacturing Practices (GSP and GMP). These measures are aimed at mainly preventing fungal growth and production of mycotoxins, both in the field and during the storage. However, under favorable weather conditions for fungal growth or poor storage conditions (such as excessive heat and moisture, insects and other pests), levels of AFs higher than the maximum permitted can be found in grain batches. Depending on the level of contamination, the contaminated materials have to be destroyed, or diverted toward biofuel production, with consequent significant economic losses for the farmers. To avoid these losses, some post-harvest decontamination/detoxification strategies have been proposed, including physical, chemical or biological methods [14,15,16,17].

Significant differences between moldy and healthy maize kernels in terms of size, shape and density have been shown. Furthermore, moldy, colored/discolored, injured, broken and damaged kernels, fine materials, as well as dust, within a contaminated batch of grains may contain very high levels of mycotoxins [18]. These materials are also ideal substrates for fungal growth because they provide readily available nutrients. A combination of cleaning technologies to efficiently remove visibly moldy, infected, broken and/or damaged kernels, as well as fine material along with smaller particles and lower density kernels, can therefore significantly reduce mycotoxin contamination in the final product. This could be done manually or by using sieves, gravimetric tables or electronic sorters. The advantage of this decontamination approach is that it reduces mycotoxin levels without producing degradation products that could be more toxic than the native mycotoxin, or any reduction in the nutritional value of the grain.

Several studies have shown that cleaning and sorting of cereals are effective processes to remove contaminated grains and significantly reducing the mycotoxin content, although the reported cleaning effects greatly vary, depending on the levels of contamination in the raw material and on the percentage of removed materials during the cleaning [19,20,21,22,23,24,25,26,27]. For instance, fumonisins levels in maize can be reduced by 29–69% by screening the fines fraction [19], whereas, in wheat cleaning, removing dust, foreign grains and other impurities before conditioning and milling reduced deoxynivalenol levels by 23–90% and T-2/HT-2 toxins by 25–80% [20,21,22,23]. Deoxynivalenol and zearalenone contamination was reduced by 73% and 79%, respectively, by removing screenings and broken kernels from maize; however, a high percentage of the total weight of the maize was removed as well [24]. Removing damaged and infected grains from the commodity showed a reduction in aflatoxin levels in maize between 40–80% [25]. In addition, manual sorting of broken and damaged kernels reduced fumonisins in maize by 84% [26], as well as AFs and fumonisins in white maize by 94–95% [27].

Manual sorting based on fluorescence using illumination with UV light (λ = 365 nm) is widely used for the reduction in aflatoxin contamination in dried figs and peanuts by removal of fluorescent material. However, this method is rarely applied in practice. A prototype system composed of belt conveyors, UV light sources, CCD cameras, optical sensors, image processing and automation software for real-time detection and automatic separation of dried figs contaminated with AFs showed a 98% success rate in the detection and separation of contaminated dried figs [28].

Optical–electronic sorting technology, developed in the 1960s, can recognize color, size, shape and structural properties allowing us to identify, by means of digital cameras, and to remove, by a short burst of compressed air, defective products and foreign materials from the production line, minimizing the loss of good products. Optical sorters are capable of removing matrix defects that are associated with high levels of mycotoxins, significantly reducing the contamination of AFs in contaminated lots of almonds [29], shelled peanuts [30,31], and of AFs and fumonisins in maize [32,33,34,35]. Current grain sorters ensure a high product flow rate that can typically sort up to 15 tons per hour.

The scale of experiments definitely has a considerable impact on the final result and pilot-scale or laboratory experiments may not always reflect what is observed in industrial processing. In addition, mycotoxin contamination in large batches is unevenly distributed and this can lead to unrealistic results when experiments are carried out at industrial level. Therefore, a reliable sampling plan should be used.

Only few studies are reported in the literature with regards to the effect of cleaning at industrial scale level in reducing mycotoxins in maize. In particular, the efficacy of maize cleaning steps on aflatoxin and fumonisin levels was evaluated in an industrial scale process aimed to assess the distribution of these mycotoxins in dry milled fractions showing a reduction in AFB_1_ and fumonisin B_1_ (FB_1_) levels by 8–57% and by 11–34%, respectively. The extent of decontamination obtained from the cleaning step depended from the levels of maize contamination [36]. In a more recent study, cleaning of maize by using a dry stoner, an intensive horizontal scourer, a vibrating aspirator and an optical sorting equipment reduced fumonisins by about 42% [37].

To our knowledge, no targeted study on the assessment of the effectiveness of cleaning/sorting combined technologies in industrial grain processing for the reduction in AFs has been carried out. The aim of this study was to evaluate the efficacy of industrial-scale cleaning solutions in reducing AFs in naturally contaminated maize. Two case studies have been performed by using three cleaning processes: (i) mechanical size separation and dust removal by aspiration, (ii) kernel separation based on density differences and (iii) optical sorting. A mass balance of AFs was carried out in order to verify the accuracy of results.

## 2. Results

### 2.1. First Case Study

The first case study was carried out in CAPA Cologna (Italy) in 2013. Four batches of maize contaminated with AFs, namely A and B (trial #1) and C and D (trial #2), were processed. A total of 15 tons of maize from each batch were cleaned by two cleaning industrial lines comprising a separator coupled to an aspirator and an optical sorter (trial #1) and a separator coupled to an aspirator, a concentrator and an optical sorter (trial #2).

Equipment was set to minimize material losses (at about 6–7%). In Table 1 the average yields of the different fractions in the two trials are reported. The incoming maize materials in the two trials showed similar characteristics in terms of broken/damaged kernels, fine and foreign materials, as highlighted by similar percentages of rejected fractions in the two processed batches.

#### 2.1.1. Trial #1 (Separator–Aspirator–Optical Sorter)

Only AFB_1_ and AFB_2_ were detected in unprocessed maize with levels of AFB_1_ of 24.2 µg/g in batch A (total aflatoxins, AFs 25.4 µg/kg) and 62.0 µg/kg in batch B (AFs 64.3 µg/kg).

After the cleaning steps (separator–aspirator–optical sorter), a significant reduction in aflatoxins levels was observed for both batch A and batch B, with a total AFB_1_ reduction of 65.3% and 78.1%, respectively (Table 2). Similar percentages of reduction were observed for total aflatoxins. The cleaning by separator plus aspirator gave a high rate of reduction (61% and 75%), showing that these steps are essential to remove highly contaminated materials (dust, fine particles, broken kernels). In fact, very high levels of AFs were found in the rejected fractions from separator and aspirator (fractions 5, 7, 8, Table 2). Similarly, high levels were also found in the rejected fraction from the optical sorter (fraction 6, Table 2) showing that the equipment was able to remove defected kernels contaminated by AFs.

#### 2.1.2. Trial #2 (Separator–Aspirator–Concentrator–Optical Sorter)

The levels of aflatoxins in batches C and D were quite similar, with 28.8 µg/kg AFB_1_ (AFs 30.2 µg/kg) in batch C and 23.5 µg/kg AFB_1_ (AFs 24.5 µg/kg) in batch D.

After the cleaning steps (separator–aspirator–concentrator–optical sorter), a significant reduction in aflatoxin levels were observed for both batch C and batch D, with AFB_1_ reductions of 84.4% and 75.8%, respectively (Table 3). A similar trend was observed for total aflatoxins. Compared to trial #1, the additional cleaning by the concentrator gave a higher rate of toxin reduction, indicating that the removal of low-density kernels provides the efficient removal of highly contaminated kernels (fraction 7, Table 3). In a similar manner to trial #1, high levels of AFs were found in the rejected fractions from the separator, aspirator and optical sorter (fractions 6, 8, 9, 10, Table 3) showing the effective role of these machines in removing contaminated materials.

### 2.2. Second Case Study

The second case study took place in 2017 in three separate steps. In the first part of the trial, the reduction in AFs that can be achieved by a mechanical separation process (separator + aspirator) using the Grain Plus LAGA (Buhler, Uzwil, Switzerland) was investigated in a cleaning plant in Nördlingen, Germany (trial #3). The cleaned material from the Grain Plus LAGA was collected and afterwards was processed by the Concentrator MTCB (Buhler, Uzwil, Switzerland) in Uzwil, Switzerland (trial #4). Finally, the cleaned product from both the Grain Plus LAGA and the Concentrator MTCB trials were cleaned by optical sorting with the SORTEX^®^ A ColorVision (Buhler, Uzwil, Switzerland) with the addition of an enhanced InGaAs camera and climate control (AC-BRBX-CCCC) in London, UK (trial #5 and trial #6). Four batches of maize of about 3 tons each with different levels of aflatoxin contamination (A1, A2, B1, B2) were used for these trials. Different conditions of the Grain Plus LAGA cleaning process were used for batches A1, B1 (normal conditions) and A2, B2 (aggressive conditions), in terms of higher sieve size at the bottom (5 mm vs. 6 mm) and flow aspiration rates (65 m^3^/min vs. 100 m^3^/min). In a similar way, normal conditions (batches A1, B1) and aggressive conditions (A2, B2), in terms of the rejection of low-density material, were applied to the concentrator, removing a larger fraction of the light product in the aggressive cleaning.

Table 4 reports the yields of the different fractions in the trials. Maize incoming materials showed different characteristics, in terms of broken/damaged kernels, fine and foreign materials, as highlighted by the different percentages of rejected fractions.

#### 2.2.1. Trial #3 (Separator–Aspirator)

Table 5 shows the levels of AFB_1_ and total aflatoxins (AFB_1_, AFB_2_, AFG_1_, AFG_2_) in the sampled fractions. The aflatoxin levels in the incoming material were in some cases (batches A1 and B1) lower than levels found in the cleaned product, probably due to non-representative samples and/or to the presence of a large amount of foreign uncontaminated materials in the incoming product. However, high levels of AFs were found in the rejected fractions from the separator and aspirator (fractions 3 and 4) showing the effective removal of contaminated materials from the unprocessed material. Aflatoxin levels in the rejected products from the normal cleaning were higher than those originating from the aggressive cleaning, showing that uncontaminated material was also removed in the aggressive cleaning process.

A more realistic level of AFs in the incoming materials was observed when the average level of AFs in the batches was calculated from the aflatoxin levels determined in the cleaned maize, broken maize (fraction rejected from separator) and dust + coarse product streams (the fraction rejected from the aspirator) using Equation (1).

(1) $$\begin{array}{ll} Calculated\;level & in\;the\;batch\;\left(µg/kg\right)\\ & =[cleaned\;(µg/kg\;)\times yield\;\left(\%\right)+broken\;\left(µg/kg\right)\times yield\;\left(\%\right)\\ & +dust\&coarse\;\left(µg/kg\right)\times yield\;\left(\%\right)]/100 \end{array}$$

From the calculated level in the batches (fraction 1, Table 6), the less-contaminated maize had aflatoxin levels under 10 µg/kg (batches A1, A2), while the more contaminated maize had aflatoxin levels close to 20 µg/kg (batches B1, B2). A similar estimation of aflatoxin levels in all the incoming materials were determined by the on-site rapidust^®^ procedure (Eurofins, Hamburg, Germany). When the calculated level was used, AF levels were lower in the cleaned maize compared to the uncleaned maize in the four trials, with the aflatoxin reduction rate ranging from 4.9% to 54.0%. 

#### 2.2.2. Trial #4 (Concentrator)

Sub-samples (~500 kg) of the cleaned material from the Grain Plus LAGA (separator + aspirator) were processed by the concentrator MTCB. Table 7 shows the levels of AFB_1_ and total aflatoxins in the sampled fractions.

After the cleaning step by the concentrator, a good reduction in AF levels was observed, with AFB_1_ reduction ranging from 45.8% to 54.8% (Table 7). A slightly higher reduction was observed for the total aflatoxins. As expected, high levels of AFs were found in the rejected fractions (fraction 3, Table 7), thus confirming the efficiency of the concentrator in removing contaminated material. No AFs were detected in samples of batch A2 (including in the rejected fraction), probably due to a sampling error during the sampling procedures. By comparing normal and aggressive cleaning conditions, no clear differences were observed in the reduction rate. However, the rejected fraction from batch B2 (aggressive conditions) had a lower AF concentration compared to that from batch B1 (normal conditions), again indicating the removal of non-contaminated products due to the aggressive cleaning.

#### 2.2.3. Trials #5 and #6 (Optical Sorter)

Sub-samples (~40 kg) of cleaned products from the Grain Plus LAGA (trial #5, sampled fractions 1a) and from the concentrator MTCB (trial #6, sampled fractions 1b) were cleaned by an optical sorting machine. The optical sorter cleaning reduced the aflatoxin concentration by 7.7–76.1%, depending on the percentage of removed fraction. It should be highlighted that rejected fractions (fraction 3, Table 8) from the optical sorter showed high aflatoxin concentrations in all trials, confirming an effective removal of defective and contaminated kernels. 

### 2.3. Mass Balance

Mass balances of AFB_1_ and total aflatoxins for the cleaning trials are reported in Table 9. The expected mass balance values in terms of recovery percentages should be close to 100%. In our experiments, mass balances in the cleaning processes, except in trial #4-batch B2, ranged from 58% to 116%. This observed variability could be related to the critical step of sampling for AFs carried out the industrial scale. The best results were observed when cleaning was performed by separator–aspirator (trial #3) and optical sorter (trials #5 and #6) as separate steps, compared to the continuous cleaning processes (trials #1 and #2). These results were probably due to the minor amounts of inputted maize materials used in the separate cleaning steps (i.e., 3 tons and 40 kg) with respect to those used in the continuous cleaning ones (i.e., 15 tons). A consistent variability of mass balance values, observed in the experiment using the concentrator, indicated a possible error in sampling. In particular, the anomalous value of trial #4-batch B2 suggests a large sampling error due to the highly heterogeneous distribution of AFs in the incoming maize material (underestimation of the aflatoxin content).

## 3. Discussion

The cleaning of cereals allows the removal of foreign materials and broken, shriveled, damaged and low-density kernels. This process is routinely carried out before the storage and/or milling of grains. Past studies that aimed to evaluate the effect of cleaning operations on the mycotoxin content in the final product have been performed at the lab and/or pilot scale, whereas studies at the industrial scale have been employed mainly to evaluate the distribution of mycotoxins in cereal milling fractions or to study the effect of food processing on mycotoxins [14,15,36,37,38,39,40,41]. Concerning maize, to our knowledge, a very limited number of studies have been carried out at the industrial-scale level, most likely due to the challenges associated with performing scientific experiments in a real industrial environment during production. The studies evaluated mainly the effect of primary processing (cleaning and milling) on the distribution of mycotoxins in the milling fractions, including AFs, zearalenone and fumonisins [36,37,38]. Only one paper reported the effect of industrial cleaning on AFs content, in two batches of maize [36]. By eliminating 3% and 6% of waste materials, a reduction of 8% and 57% was observed with maize contaminated at 3.6 µg/kg and 91.1 µg/kg, respectively. The cleaning operations included sieving (winnower), separation (dry stoner), intensive scouring and aspiration [36]. In our study, we explored the efficacy of different pieces of industrial-scale cleaning equipment on the reduction in the AFs in maize. These included a sieving machine combined with an aspirator, a concentrator to separate high-density, mixed, and low-density material and an optical sorter. This equipment was used in-line (continuous processing) or separately (sequential batch processing).

The understanding of both continuous and batch processing is of great importance in order to gain a complete picture of the mycotoxin reduction potential of each system component, and ultimately to be able to give recommendations for practical implementation in grain processors. For example, significant interaction effects are expected, where the first order effects are caused by an overlap of the rejected fractions (e.g., a small grain removed by sieving might also have anomalous optical properties detected by an optical sorter) and the second order effects may be attributed to a change in sorting/separation performance due to a change in grain throughput (tons/h). The combination of the tested cleaning machines allowed a total aflatoxin removal between 65–84% of maize contaminated at levels ranging from 25 to 65 µg/kg, showing a greater reduction and indicating more efficient cleaning than that reported by Pietri et al. [35]. Additionally, an overall reduction in AFs ranging from 55% to 94% can be estimated by combining the results from the separate cleaning steps. This range was obtained by considering the lowest and the highest reduction values in each step.

Ideally, the percent of rejected fraction should be a compromise between acceptable economic losses and acceptable mycotoxin concentration in the final product. Generally, a value of 5% is considered acceptable however this may differ in specific situations and grain contamination patterns. In our study, machines were set to higher percent values of rejected fractions in order to achieve acceptable percentages of AF reduction—for example, the total percentages of rejected product in the second case study were quite high (ranging from 7% to 27%) compared to the first case study (6–7%) because different conditions (normal and aggressive conditions) were tested. The percent of rejected fraction should be optimized, preferably in pilot plants. The choice of the equipment or combination of equipment to be used for cleaning and the percentage of the rejected fractions to be set depend on the quality of the raw material and anticipated mycotoxin contents.

Generally, typical cleaning plants are equipped with sieving equipment combined with an aspirator. Our results show that the dust and broken kernels contained very high aflatoxin levels, which were removed by sieving machines using aspiration, which also removes coarser foreign materials. The percentage of rejected fraction setting depends on the raw material. This pre-cleaning step is necessary prior to storage and is routinely carried out at a grain collection point, however, depending on the level of mycotoxin contamination, it may be not sufficient for obtaining maize with mycotoxin content below legal limits. Therefore, advanced grain cleaning is necessary before further processing. A concentrator, which allows kernel separation based on density differences, and an optical sorter, which allows removal of grains with visual defects, have both been shown to be effective tools for reducing AFs contamination as they increase the probability of removing contaminated versus uncontaminated grains. A detailed analysis, including total processing volume and expected contamination levels, is required for grain processors to ensure economic viability and to offset initial investment costs. The availability of public funding initiatives for increasing agricultural investments could positively influence the interest of grain processors in investing in these grain-cleaning devices.

The first case study reported in the present manuscript is an example of how an actual cleaning line using a combination of separator–aspirator–concentrator–optical sorter allowed a grain seller to recover inferior maize destined to biofuels, and instead diverted it back toward feed or food. In 2013, aflatoxin levels were extremely high in maize in Italy and a grain seller had to cope with several tons of maize with a level of AFB_1_ higher than 20 µg/kg, which is the regulated limit for complete feedstuffs for pigs and poultry in the EU [42]. The same combination of cleaning equipment allowed the removal of the contaminated fractions and recovered the maize for the feed industry. This has helped to reduce economic losses, while ensuring the safety of the feed products [42]. Additionally, optical sorters are commonly used in industrial plants to remove defected kernels, kernels with color variations and foreign materials by means of pneumatic ejectors and real-time image processing [43,44,45,46,47]. They are commonly applied to nuts, seeds, grains, coffee and legumes, providing superior quality products. Pearson et al. showed that a high-speed sorter (throughput of 7000 kg/h) using selected filters was able to reduce aflatoxin levels by 81% in naturally contaminated maize at 53 µg/kg by rejecting 5% of grains [32]. In our study, a similar aflatoxin reduction (i.e., about 80%) was reached by rejecting up to 8% of grains although a higher throughput was used in the trials (i.e., 15000 kg/h).

It should also be noted that appropriate sampling is a precondition for achieving accurate results and for drawing reliable conclusions, mainly when experiments are carried out at industrial scale. Although in this study sampling was performed according to guidelines reported in the Commission Regulation No. 401/2006 [48] and slurry mixing was used to minimize subsampling errors, our results show that sampling is the most critical step when experiments are carried out at industrial scale for AFs, which are well-known for their heterogeneous distribution in the matrix. This conclusion agrees with Brera and colleagues’ assessment, “a sampling error should always be taken in account, even if the sampling procedure has been performed according to the European Directive” [38]. However, our study provided strong evidence that high levels of AFs are found in all the rejected fractions indicating that the cleaning equipment used, is able to remove materials highly contaminated with AFs. We would like to stress that due to the seasonal and regional variability of fungal contaminants and resulting impact on the properties of the grain, the levels of AF contamination in the various fractions might vary.

## 4. Conclusions

This study shows that a cleaning line, combining both mechanical and optical sorting technologies, can provide an efficient solution for a significant reduction in aflatoxin contamination in maize. Some cornerstones of successful aflatoxin reduction in maize can be highlighted, as follows: (i) removal of dust, coarse, fine, and low-density impurities; (ii) removal of grain with a low bulk density; (iii) sorting of discolored and defective grains. The results also highlighted a criticism in the sampling plan for AFs, suggesting that a higher number of incremental samples should be collected, with respect to those fixed by the European Commission. Overall results show that an accurate maize cleaning by appropriate cleaning equipment could allow us to convert biomass/feed quality grains to feed/food quality grains with significant economic gains for the farmers. This outcome could be particularly useful in critical crop seasons in which the levels of AFs in cereals intended for human or animal consumption might exceed the maximum permitted levels.

## 5. Materials and Methods 

### 5.1. Samples and Cleaning Processes

**Figure 1:**
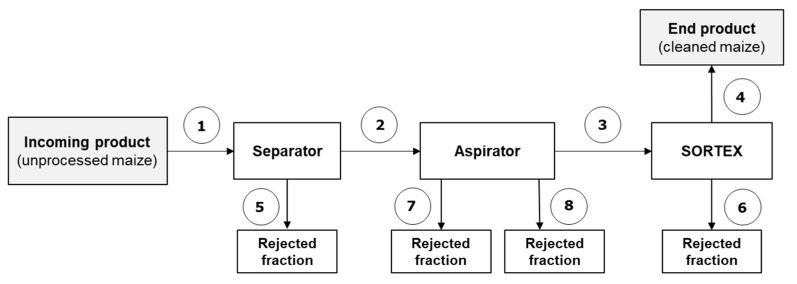
Scheme of the industrial cleaning line and sampling points (numbered)-Trial #1 (Separator–aspirator-SORTEX, in-line). Sampling points 1, 2, 3, 4, 5, 6: dynamic sampling; 7, 8: static sampling.

**Figure 2:**
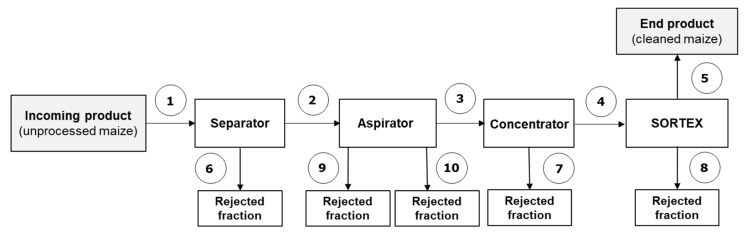
Scheme of the industrial cleaning line and sampling points (numbered)-Trial #2 (separator–aspirator–concentrator–SORTEX, in-line). Sampling points 1, 2, 3, 4, 5, 6, 7, 8: dynamic sampling; 9, 10: static sampling.

**Figure 3:**
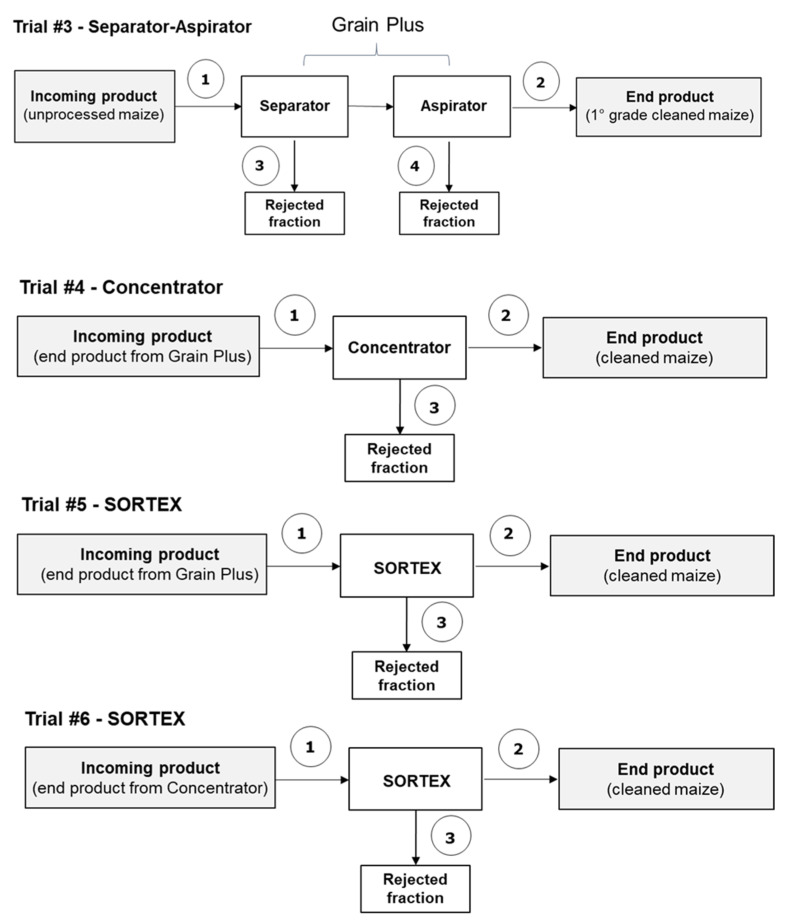
Scheme of the industrial cleaning lines and sampling points (numbered) – Trials #3-6 (separator–aspirator–concentrator–SORTEX, separate steps). Sampling points 1, 2, 3: dynamic sampling; 4: static sampling.

Schemes of the industrial cleaning lines and sampling points are shown in Figure 1, Figure 2 and Figure 3. In the first case study, four batches of maize naturally contaminated with AFs, namely A and B (trial #1) and C and D (trial #2), were processed by two continuous cleaning industrial lines comprising a separator with aspirator (high capacity multilayer sifter), a concentrator (MTCB, Buhler AG, Switzerland) and an optical sorter (SORTEX A5 BRBX, Buhler AG, City, Switzerland). The separator and aspirator machines remove broken and fine kernels, as well as coarse and light impurities. The concentrator MTCB has been developed for the classification of granular materials into three different fractions: high-density, mixed, and low-density material. The low-density material is generally removed. Grain cleaning by SORTEX is based on optical sorting. Foreign bodies and kernels with visual defects are removed. Specifically, in trial #1, the process line included separator, aspirator and optical sorter (Figure 1); in trial #2 the process line included separator, aspirator, concentrator and optical sorter (Figure 2). The plant, located in CAPA Cologna (Northern Italy), was able to process 15 tons/h of raw maize.

In the second case study, four batches of maize (A1, A2, B1, B2) naturally contaminated with AFs (about 3 tons each) were processed by separate steps. First, mechanical separation with the Grain Plus (Bühler AG, Uzwil, Switzerland), a machine combining aspiration and sieve cleaning into one machine, was performed in a cereal cleaning plant located in Nördlingen (Southern Germany). Different equipment conditions were used to process the four batches of maize material (i.e., batches A1, B1—normal conditions; batches A2, B2—aggressive conditions) in terms of sieve size at the bottom (5 mm vs. 6 mm) and flow aspiration rates (65 m^3^/min vs. 100 m^3^/min) (trial #3, Figure 3). Afterwards, aliquots (~500 kg) of cleaned material from the Grain Plus separator were further processed at Buhler AG (Uzwil, Switzerland) with the MTCB concentrator (Bühler AG, Uzwil, Switzerland) based on density differences using two different conditions (batches A1, B1—normal rejection of low-density material; batches A2, B2—high rejection of low-density material) (trial #4, Figure 3). Finally, aliquots (~40 kg) of cleaned products from both Grain Plus and concentrator trials were optically sorted by the SORTEX A ColorVision with added Enhanced InGaAs camera and climate control (Bühler AG, Uzwil, Switzerland) in London, UK (trials #5 and #6, Figure 3). The SORTEX machine separated the kernels in two fractions called “accept” (end product) and “reject” (rejected fraction).

Fractions were sampled as reported in Section 5.2 and analyzed for aflatoxin B1 and total aflatoxin content, as reported in Section 5.3.

### 5.2. Sampling

Sampling was performed according to the European Commission Regulation N. 401/2006 for sampling method of cereals and cereal products by taking into account the weight of processed maize and rejected fractions in each step [48]. Incremental samples (from five to 60) were dynamically sampled from opening slits of the plants/machines. Sub-samples of 100–300 g were collected for 1 h at regular intervals. Only for the rejected fractions from the aspirator was a static sampling procedure adopted by randomly sampling the incremental samples (about 300–500 g). The number of incremental samples and the weight of the aggregate samples to be submitted for analysis are reported in Table 10. Sampling points are indicated in Figure 1, Figure 2 and Figure 3. All sampled fractions were analyzed for aflatoxin B1 and total aflatoxin content, as reported in Section 5.3.

In addition, the sampling of dust was performed during the whole download of the unprocessed maize batches to be cleaned by the Grain Plus machine (trial #3) using the rapidust^®^ system (Eurofins, Hamburg, Germany) for on-site sampling and the analysis of mycotoxins in grains. This system allows for the reliable calculation of AF contamination in the grain based on concentrations determined in the respective dust samples.

### 5.3. Aflatoxin Analysis

Analyses of aflatoxins (AFB_1_, AFB_2_, AFG_1_ and AFG_2_) were performed according to the AOAC Official Method No. 2005.008 for the determination of aflatoxins in corn, raw peanuts and peanut butter, with minor modifications. First, in order to minimize subsampling errors, aggregate samples were mixed with slurry, i.e., all sampled fractions were vigorously mixed with water for 10 min by using the Silverson EX high shear mixer equipped with a general purpose disintegrating head (Silverson Machines Ltd, Waterside, Chesham, UK). A matrix:water ratio of 1:1 or 1:1.5 (w/w) was used, depending on the fraction. In the case of fractions up to 1 kg (including recovery experiments), an Ultra Turrax IKA T25 (IKA Werke GmbH & Co. KG., Staufen, Germany) was used for the preparation of slurries. Aliquots of slurry (50 g) were extracted with methanol water (70:30, v/v) by blending at high speed for 2 min (Sorvall Omnimixer). The extracts were filtered through Whatman No. 1 filter paper (Whatman, Maidstone, UK) and 10 mL of filtered extract was diluted with 40 mL of distilled water and mixed. The diluted extract was filtered through the glass microfiber filter Whatman GF/A (Whatman, Maidstone, UK). Ten mL of the filtered diluted extract was passed on the AflaTest immunoaffinity column (VICAM, a Waters Business, Milford, MA, USA) at a rate of about 1 drop/s. The immunoaffinity column was washed with 2 × 5 mL water at a flow rate of 1–2 drops/s. Aflatoxins were eluted with 1 mL methanol in a 4 mL silanized vial. The eluted extract was gently dried under a nitrogen stream at about 40 °C and reconstituted with 500 μL of LC mobile phase. An aliquot of reconstituted extract (20 µL) was injected into the chromatographic apparatus by full loop injection. The LC apparatus consisted of the Acquity UPLC (Waters, Milford, MA, USA) equipped with a fluorescence detector set at (_ex_ = 365 nm, _em_ = 435 nm. The mobile phase consisted of a isocratic mixture of water:acetonitrile:methanol (64:18:18, v/v/v) at a flow rate of 0.4 mL/min. The analytical column was the Acquity UPLC BEH C18 (100 × 2.1 mm, 1.7 µm). With these conditions, AFs eluted in the order AFG_2_, AFG_1_, AFB_2_ and AFB_1_ within 4 min. The quantification of AFs was performed by measuring peak areas at AFs retention time, and comparing them with the relevant calibration curves.

Limits of Detection (LODs), based on a signal-to-noise ratio of 3:1, were 0.3 µg/kg for AFB_1_ and AFG_1_ and 0.1 µg/kg for AFB_2_ and AFG_2_. Recoveries from maize ranged from 84% to 96% with relative standard deviations (RSDs) <7% (spiking levels: 5–50 µg/kg, triplicate measurements).

### 5.4. Mass Balance

The mass balance (%) was calculated with respect to AFB_1_, as [∑ absolute amount of AFB_1_ in all rejected fractions plus absolute amount of AFB_1_ in the final cleaned fraction / absolute amount of AFB_1_ in the incoming product (unprocessed maize)] × 100. Similar calculations were performed for total aflatoxins. Information about the mass loss (%) was provided, taking in account the technical specifications of the plants and from previous measurements carried out during processing before starting with the trials (i.e., by considering the flow rate and measuring the weight of rejected fractions after 10 min cleaning).

## Figures and Tables

**Table 1:** Yields (%) of maize-cleaning fractions—first case study.

Maize-Cleaning Fraction	Trial #1	Trial #2
Unprocessed maize	100	100
Rejected fraction from separator Rejected fraction from aspirator	0.83 0.76	0.83 0.32
Rejected fraction from concentrator		3.49
Rejected fraction from optical sorter	4.59	2.00
Cleaned maize	93.82	93.36

**Table 2:** Effect of industrial-scale continuous cleaning line (separator–aspirator–optical sorter) on aflatoxin contents in sampled fractions (Trial #1).

Batch	Sampled Fraction ^1^	AFB_1_ (µg/kg)	AFB_1_ Reduction (%)	AFs ^2^ (µg/kg)	AFs Reduction (%)
A	1	24.2	65.3	25.4	65.3
	2	17.3		17.9	
	3	9.5		9.8	
	4	8.4		8.8	
	5	109.1		114.1	
	6	76.0		79.5	
	7	165.0		170.6	
	8	319.6		333.0	
B	1	62.0	78.1	64.3	77.7
	2	28.6		29.8	
	3	15.8		16.7	
	4	13.5		14.3	
	5	110.7		115.5	
	6	468.4		489.4	
	7	195.6		203.2	
	8	309.8		322.5	

^1^ #1 Unprocessed maize (incoming product); #2 cleaned maize from separator; #3 cleaned maize from aspirator; #4 cleaned maize from optical sorter (end product); #5 rejected fraction from separator; #6 rejected fraction from optical sorter; #7, 8 rejected fractions from aspirator; ^2^ Sum of aflatoxin B_1_ and B_2_ (AFB_1_ and AFB_2_).

**Table 3:** Effect of industrial-scale continuous cleaning line (separator–aspirator–concentrator–optical sorter) on aflatoxin contents in sampled fractions (Trial #2).

Batch	Sampled Fraction ^1^	AFB_1_ (µg/kg)	AFB_1_ Reduction (%)	AFs ^2^ (µg/kg)	AFs Reduction (%)
C	1	28.8	84.4	30.2	84.4
	2	11.4		11.8	
	3	12.7		13.2	
	4	5.4		5.6	
	5	4.5		4.7	
	6	120.8		125.8	
	7	264.1		274.6	
	8	327.2		340.3	
	9	182.0		189.1	
	10	328.8		341.2	
D	1	23.5	75.8	24.5	75.6
	2	10.7		11.1	
	3	6.3		6.4	
	4	5.4		5.5	
	5	5.7		6.0	
	6	127.1		132.1	
	7	159.9		165.7	
	8	244.1		265.4	
	9	164.9		170.9	
	10	306.9		318.7	

^1^ #1 Unprocessed maize (incoming product); #2 cleaned maize from separator; #3 cleaned maize from aspirator; #4 cleaned maize from concentrator; #5 cleaned maize from optical sorter (end product); #6 rejected fraction from separator; #7 rejected fraction from concentrator; #8 rejected fraction from optical sorter; #9, 10 rejected fractions from aspirator; ^2^ Sum of AFB_1_ and AFB_2_.

**Table 4:** Yields (%) of maize-cleaning fractions—second case study.

Trial	Maize-Cleaning Fraction	Batch			
		A1	A2	B1	B2
# 3	Unprocessed maize	100	100	100	100
	Rejected fraction from separator	1.7	4.3	6.8	8.8
	Rejected fraction from aspirator	0.2	1.1	0.7	2.2
	Cleaned maize	98.1	94.6	92.5	89.0
# 4	Cleaned maize from separator + aspirator	100	100	100	100
	Rejected fraction from concentrator	3.0	17.6	2.4	12.2
	Cleaned maize	97.0	82.4	97.6	87.8
# 5	Cleaned maize from separator + aspirator	100	100	100	100
	Rejected fraction from optical sorter	5.1	3.8	7.9	5.6
	Cleaned maize	94.9	96.2	92.1	94.4
# 6	Cleaned maize from concentrator	100	100	100	100
	Rejected fraction from optical sorter	4.3	4.1	6.9	5.4
	Cleaned maize	95.7	95.9	93.1	94.6

**Table 5:** Effect of industrial-scale cleaning by separator–aspirator on aflatoxin contents in sampled fractions (Trial #3).

Batch	Sampled Fraction ^1^	AFB_1_ (µg/kg)	AFB_1_ Reduction (%)	AFs ^2^ (µg/kg)	AFs Reduction (%)
A1	1	n.d. ^3^	-	n.d.	-
	2	0.8		1.5	
	3	39.4		44.2	
	4	142.4		222.0	
A2	1	15.3	58.9	19.1	64.4
	2	6.3		6.8	
	3	21.2		32.1	
	4	80.1		110.7	
B1	1	15.9	-	17.6	-
	2	19.0		29.7	
	3	33.8		41.0	
	4	124.8		139.5	
B2	1	28.3	30.7	30.4	30.6
	2	19.6		21.1	
	3	22.1		31.1	
	4	94.9		105.9	

^1^ #1 Unprocessed maize (incoming product); #2 cleaned maize from separator + aspirator (end product); #3 rejected fraction from separator; #4 rejected fraction from aspirator; ^2^ AFs: sum of AFB_1_, AFB_2_, AFG_1_, AFG_2_; ^3^ n.d. = not detected (Limit of Detection (LOD): AFB_1_, AFG_1_: 0.3 µg/kg; AFB_2_, AFG_2_: 0.1 µg/kg).

**Table 6:** Aflatoxin levels in sampled fractions and relevant reduction values, with calculated input values according to Equation (1) (Trial #3).

Batch	Sampled Fraction ^1^	AFB_1_ (µg/kg)	AFB_1_ Reduction (%)	AFs ^2^ (µg/kg)	AFs Reduction (%)
A1	1 2	1.7 0.8	54.0	2.7 1.5	43.8
A2	1 2	7.8 6.3	18.7	9.0 6.8	24.6
B1	1 2	20.8 19.0	8.4	31.2 29.7	4.9
B2	1 2	21.5 19.6	8.7	23.9 21.1	11.5

^1^ #1 Incoming unprocessed maize; #2 cleaned maize from separator + aspirator (end product); ^2^ AFs: sum of AFB_1_, AFB_2_, AFG_1_, AFG_2_.

**Table 7:** Effect of industrial-scale cleaning by concentrator on aflatoxin contents in sampled fractions (Trial #4).

Batch	Sampled Fraction ^1^	AFB_1_ (µg/kg)	AFB_1_ Reduction (%)	AFs ^2^ (µg/kg)	AFs Reduction (%)
A1	1	2.4	45.8	2.5	48.0
	2	1.3		1.3	
	3	19.1		19.5	
A2	1	n.d. ^3^	-	n.d.	-
	2	n.d.		n.d.	
	3	n.d.		n.d	
B1	1	11.5	54.8	15.4	64.9
	2	5.2		5.4	
	3	73.4		77.0	
B2	1	2.9	48.3	3.2	53.1
	2	1.5		1.5	
	3	110.9		117.6	

^1^ #1 Cleaned maize from separator + aspirator (incoming product); #2 Cleaned maize from concentrator (end product); #3 rejected fraction from concentrator (light fraction); ^2^ AFs: sum of AFB_1_, AFB_2_, AFG_1_, AFG_2_; ^3^ n.d. = not detected (LOD: AFB_1_, AFG_1_: 0.3 µg/kg; AFB_2_, AFG_2_: 0.1 µg/kg).

**Table 8:** Effect of industrial-scale cleaning by optical sorter on aflatoxin contents in sampled fractions (Trials #5 and #6).

Batch	Trial	Sampled Fraction ^1^	AFB_1_ (µg/kg)	AFB_1_ Reduction (%)	AFs ^2^ (µg/kg)	AFs Reduction (%)
A1		1	5.7	50.9	6.2	50.0
	#5	2	2.8		3.1	
		3	58.5		62.6	
		1	1.3	7.7	1.5	13.3
	#6	2	1.2		1.3	
		3	4.9		5.2	
A2		1	0.8	25.0	0.8	25.0
	#5	2	0.6		0.6	
		3	5.1		5.3	
		1	7.0	11.4	7.9	10.1
	#6	2	6.2		7.1	
		3	24.9		26.4	
B1		1	9.0	62.2	9.7	60.8
	#5	2	3.4		3.8	
		3	74.1		78.0	
		1	11.7	75.2	13.4	76.1
	#6	2	2.9		3.2	
		3	130.7		151.0	
B2		1	28.7	31.4	31.6	33.5
	#5	2	19.7		21.0	
		3	180.2		209.8	
		1	7.0	42.9	7.8	46.2
	#6	2	4.0		4.2	
		3	60.2		69.9	

^1^ #1 Cleaned maize from separator + aspirator (trial #5) or concentrator (trial #6) (incoming product); #2 cleaned maize from optical sorter (end product); #3 rejected fraction from optical sorter; ^2^ AFs: sum of AFB_1_, AFB_2_, AFG_1_, AFG_2_.

**Table 9:** Aflatoxin B_1_ and total aflatoxins mass balance (%).

Trial	Batch	AFB_1_ (%)	AFs (%)
#1 Separator–aspirator–optical sorter	A	58	58
	B	60	60
#2 Separator–aspirator–concentrator–optical sorter	C	76	75
	D	75	76
#3 Separator–aspirator	A1	102	99
	A2	99	100
	B1	100	100
	B2	100	100
#4 Concentrator	A1	76	74
	A2	-^1^	-^1^
	B1	59	58
	B2	512	490
#5 Optical sorter	A1	99	99
	A2	96	97
	B1	100	100
	B2	100	100
#6 Optical sorter	A1	103	98
	A2	100	100
	B1	100	100
	B2	100	99

^1^ - not calculated, mass balance was not calculated because aflatoxins were not detected in the fractions (see Table 7).

**Table 10:** Number of incremental samples of sampled fractions, according to the Commission Regulation N. 401/2006 [48].

Trial	Sampled Fraction ^1^	Fraction Weight (Tons)	Number of Incremental Samples	Aggregate Sample Weight (Kg)
#1	1, 2, 3, 4	<10–≤20	60	6–10
	5	>0.5–≤1.0	10	1–2
	6, 7, 8	>0.05–≤0.5	5	1–2
#2	1, 2, 3, 4, 5	<10–≤20	60	6–10
	7	>0.5–≤1.0	10	1–2
	6, 8	>0.05–≤0.5	5	1–2
	9, 10	≤0.05	3	1–1.5
#3–6	1, 2	>1.0–≤3.0	20	2–4
	3	>0.05–≤0.5	5	1–2
	4	≤0.05	3	1–1.5

^1^ See Figure 1, Figure 2 and Figure 3.
